# Role of Fc Core Fucosylation in the Effector Function of IgG1 Antibodies

**DOI:** 10.3389/fimmu.2022.929895

**Published:** 2022-06-30

**Authors:** Josée Golay, Alain E. Andrea, Irene Cattaneo

**Affiliations:** ^1^ Center of Cellular Therapy "G. Lanzani", Division of Hematology, Azienda Socio Sanitaria Territoriale Papa Giovanni XXIII, Bergamo, Italy; ^2^ Laboratoire de Biochimie et Thérapies Moléculaires, Faculté de Pharmacie, Université Saint Joseph de Beyrouth, Beirut, Lebanon

**Keywords:** therapeutic antibodies, IgG, N-glycan, fucosylation, ADCC, NK cells, virus, humoral response

## Abstract

The presence of fucose on IgG1 Asn-297 N-linked glycan is the modification of the human IgG1 Fc structure with the most significant impact on FcɣRIII affinity. It also significantly enhances the efficacy of antibody dependent cellular cytotoxicity (ADCC) by natural killer (NK) cells *in vitro*, induced by IgG1 therapeutic monoclonal antibodies (mAbs). The effect of afucosylation on ADCC or antibody dependent phagocytosis (ADCP) mediated by macrophages or polymorphonuclear neutrophils (PMN) is less clear. Evidence for enhanced efficacy of afucosylated therapeutic mAbs *in vivo* has also been reported. This has led to the development of several therapeutic antibodies with low Fc core fucose to treat cancer and inflammatory diseases, seven of which have already been approved for clinical use. More recently, the regulation of IgG Fc core fucosylation has been shown to take place naturally during the B-cell immune response: A decrease in α-1,6 fucose has been observed in polyclonal, antigen-specific IgG1 antibodies which are generated during alloimmunization of pregnant women by fetal erythrocyte or platelet antigens and following infection by some enveloped viruses and parasites. Low IgG1 Fc core fucose on antigen-specific polyclonal IgG1 has been linked to disease severity in several cases, such as SARS-CoV 2 and Dengue virus infection and during alloimmunization, highlighting the *in vivo* significance of this phenomenon. This review aims to summarize the current knowledge about human IgG1 Fc core fucosylation and its regulation and function *in vivo*, in the context of both therapeutic antibodies and the natural immune response. The parallels in these two areas are informative about the mechanisms and *in vivo* effects of Fc core fucosylation, and may allow to further exploit the desired properties of this modification in different clinical contexts.

## Introduction

IgGs are among the most abundant proteins in the circulation (700-1600 mg/dl in healthy adults), and specific IgGs are induced in response to infection, endogenous or allogeneic challenges, or by vaccination. Different IgG subclasses are found in man, which are very similar structurally but have distinct functions due to their differential binding to FcɣRs, complement components as well as other proteins. About 60% of plasma IgG is IgG1, 32% IgG2 and 4% each IgG3 and IgG4 in humans (1). IgGs are glycoproteins and their glycosylation pattern can change during time, due to age, diseases or environmental factors (2, 3).

Therapeutic monoclonal antibodies (mAbs) have emerged as an important therapeutic option in cancer since the approval in 1997 of the anti-CD20 antibody rituximab for the treatment of B-non Hodgkin's lymphoma (B-NHL). Since then, antibodies directed against different antigens expressed by cancer, immune cells or infectious agents have been developed to treat a variety of diseases. Indeed, so far, over 130 antibodies have been approved by the US and EU Drug Agencies, with 45% for oncological disorders, 27% for immune- or inflammation-related conditions and the rest for infectious or other diseases (4).

Most unconjugated therapeutic mAbs are IgG1 or in some cases IgG4 or IgG2. This is because the human IgG1 Fc moiety interacts efficiently with activating FcɣRs (FcɣRI, IIA, IIC, IIIA and IIIB), expressed on the surface of immune cells (1, 5). This interaction leads to antibody-dependent cellular cytotoxicity (ADCC) by NK cells (mostly *via* FcɣRIIIA, CD16A) (6, 7), antibody dependent phagocytosis (ADCP) by macrophages (mostly through FcɣRI, CD64 and to some extent FcɣRIIA, CD32A) (8–12) and ADCC/ADCP by polymorphonuclear neutrophils (PMN)(mostly *via* FcɣRIIA, CD32A) (13–15). IgG1 also interacts with FcɣRIIIB (CD16B), a GPI-linked molecules lacking activating domain, highly expressed by PMN and involved in PMN mediated ADCC and ADCP, but whose role may be either activating or inhibiting, perhaps depending on stimulus (13–16). Immune cell activation *via* FcɣRs also induces the release of cytokines and chemokines that may cooperate in eliminating the target cells but also induce unwanted side-effects (17). Finally the Fc region of human IgG1 can bind to the first component of the complement cascade C1q and activate the classical pathway of complement which may lead to cell lysis and death through complement dependent cytotoxicity (CDC), as well as phagocytosis by macrophages and PMN through complement receptors on these cells (18). Therefore, many therapeutic antibodies against cancer cells or other targets are of the IgG1 isotype to allow activation of a panoply of immune-mediated mechanisms, many of which rely on FcɣRs.

When the activation of the immune system is not desired, for example when a therapeutic antibody is required only to neutralize the antigen, such as a growth factor or checkpoint inhibitor, then the human IgG4 or IgG2 subclasses are often chosen, because they do not interact efficiently with FcɣRs or with C1q. The more recent human IgG4 formats include a mutation in Fc (S228A) to avoid Fab arm exchange, a natural phenomenon that leads to IgG4 instability (19).

Over the last 10-15 years, various modifications of antibody structures have been introduced to increase the efficacy of therapeutic mAbs *in vitro* and *in vivo*: these include extensively modified Abs with additional effector functions, such as bispecific antibodies (bsAbs), antibody-drug conjugates (ADCs) and fusion proteins carrying for example cytokines (17, 20). Less dramatic modifications of therapeutic mAbs include the introduction of point mutations in the Fc domain, as well as modification of Fc N-linked glycans that modulate IgG binding to FcɣRs and therefore enhance or abolish Fc mediated immune activation (ADCC, ADCP and/or CDC) (17, 20).

In this paper, we will summarize the knowledge gained about the role of IgG1 N-glycan core fucosylation in the *in vitro* and *in vivo* functions of IgG1 antibodies. Ig isotypes or subclasses other than IgG1 bear N-glycans, but less is known about the role of core Fc fucosylation in their case and these will not be further discussed here. Interestingly, the studies on the role of Fc core fucose in therapeutic IgG1 mAbs has facilitated the detection and understanding of the significance of this modification, observed during the polyclonal IgG1 response to some infectious agents, alloimmunization and in some autoimmune conditions. The knowledge on these aspects will therefore also be summarized and discussed.

## The IgG N-Linked Glycans

Human IgGs are glycosylated proteins with a complex and variable glycosylation pattern. An important and extensively studied N-glycosylation site is present at conserved Asparagine 297 (Asn 297) in the CH2 domain, that interacts with FcɣRs. 20-30% of IgGs also bear N-glycans on Fab arms (21). Although there are reports of functional effects of different Fab N-glycosylation patterns in some antibodies (22), these are likely to be mostly antibody specific (23). Detailed structural studies of several commercial therapeutic mAbs has revealed that although Fab interacts with FcɣRIIIA and stabilizes the Fc-FcɣRIIIA binding, Fab fucosylation has a limited effect on the affinity of IgG for FcɣRIIIA (23, 24). The modulation of Fab fucosylation will therefore not be further discussed here.

**Figure 1:**
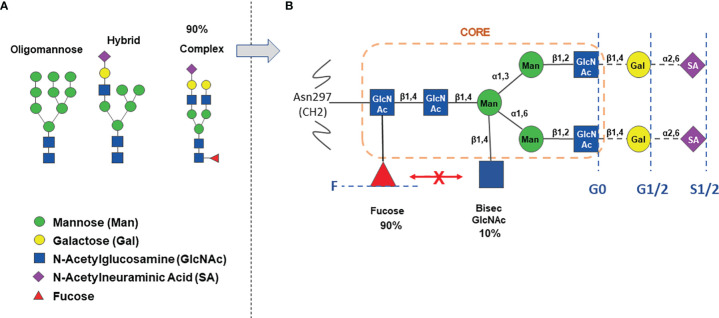
The IgG Asn-297 N-glycan heterogeneity. Panel **(A)** major types of N-glycosylation observed in IgG. Panel **(B)** detail of complex type glycosylation with percentage of circulating IgG containing either fucose or bisecting N-Acetylglucosamine. The orange circle indicates the core structure. The blue broken lines and text indicate the heterogeneity of complex N-glycans, carrying either no Gal (G0), 1-2 Gal (G1/2), 1-2 Sialic acids (S1/2), with (F) or without core α-1,6-fucose.

The IgG Asn 297 N-glycans show a high degree of microheterogeneity, and they can be grouped in oligomannose, hybrid or complex type, the latter being the most abundant (about 90%) in IgG, either circulating or produced by cell lines *in vitro* (25) (**Figure 1A**). The presence of Fc N-glycan induces in general a more open structure compared to aglycosylated IgG, favors binding to activating FcɣRs and promotes antibody stability *in vitro* and *in vivo* (1, 26). The complex type N-glycosylation itself shows microheterogeneity: whereas it always contains a heptaglycan biantennary core structure (four GlcNAc and three mannose residues), the core can bear an additional bisecting GlcNAc (in about 10% of IgGs), and 1 or 2 galactose residues (in 35% and 15% of IgG, respectively) and 1-2 terminal N-acetylneuraminic acid (sialic acid, SA), on 10-15% of IgGs (**Figure 1B**). Finally, an α-1,6 fucose residue (core fucose) is present in 90% of complex type IgG N-glycans (**Figure 1B**). Interestingly the presence of bisecting GlcNAc inhibits α-1,6 core fucosylation due to steric hindrance and therefore IgGs generally contain either a bisecting GlcNAc or a core fucose residue, although some IgGs may have both bisecting GlcNAc and fucose (2, 27–29). IgGs are composed of least 30 glycovariants, to which specific abbreviations have been assigned: G0 (no Gal residue), G1 (1 Gal), G2 (2 Gals), F (fucose) etc (**Figure 1B**) **(** 2, 27–29).

## The Biosynthesis of Human IgG N-Linked Glycans

**Figure 2:**
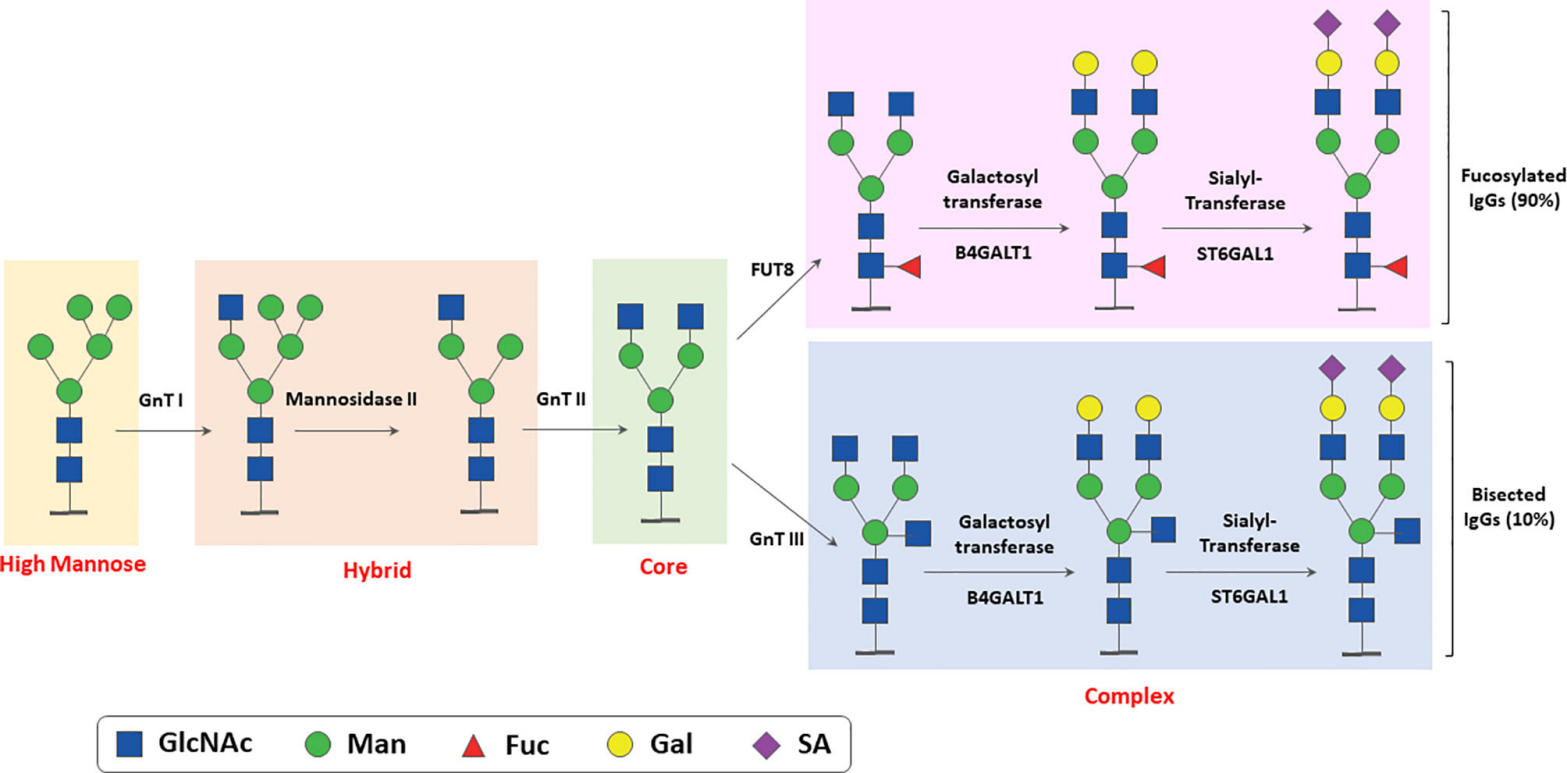
Simplified scheme of enzymatic reactions involved in N-linked glycosylation of IgG. The major glycosylation steps and enzymes involved in N- linked glycosylation of IgGs taking place in ER and Golgi are shown. GnT, N-Acetyl glucosamine transferase; FUT, Fucosyl transferase; Man, Mannose; Gal, Galactose; GlcNAcm N-acetylglucosamine; SA, sialic acid (N-Acetylneuraminic Acid).

N-glycosylation is a multi-step enzyme-mediated biochemical process. IgG N-glycan biosynthesis starts in the endoplasmic reticulum with the addition of a pyrophosphate-dolichol precursor (Dol-P, Glc3Man9GlcNAc2) to the Asn 297 N-glycosylation site of IgG. This structure is then trimmed by glucosidases and mannosidases, as the process moves to the Golgi, leading to the formation of high mannose, hybrid and the complex types N-glycans (26, 30–33). The main enzymatic reactions taking place in the Golgi are summarized in **Figure 2**. The stable overexpression or reduction/inhibition of some of these enzymes in antibody producing cell lines have been used to modify IgG glycan composition and perform structure-function studies of N-glycan microheterogeneity ( (31); and see below). The structure of N-linked and other IgG glycans and glycosylation pathways have already been extensively reviewed by others (30, 32, 34, 35) and we refer the readers to these more complete descriptions of the process.

**Figure 3:**
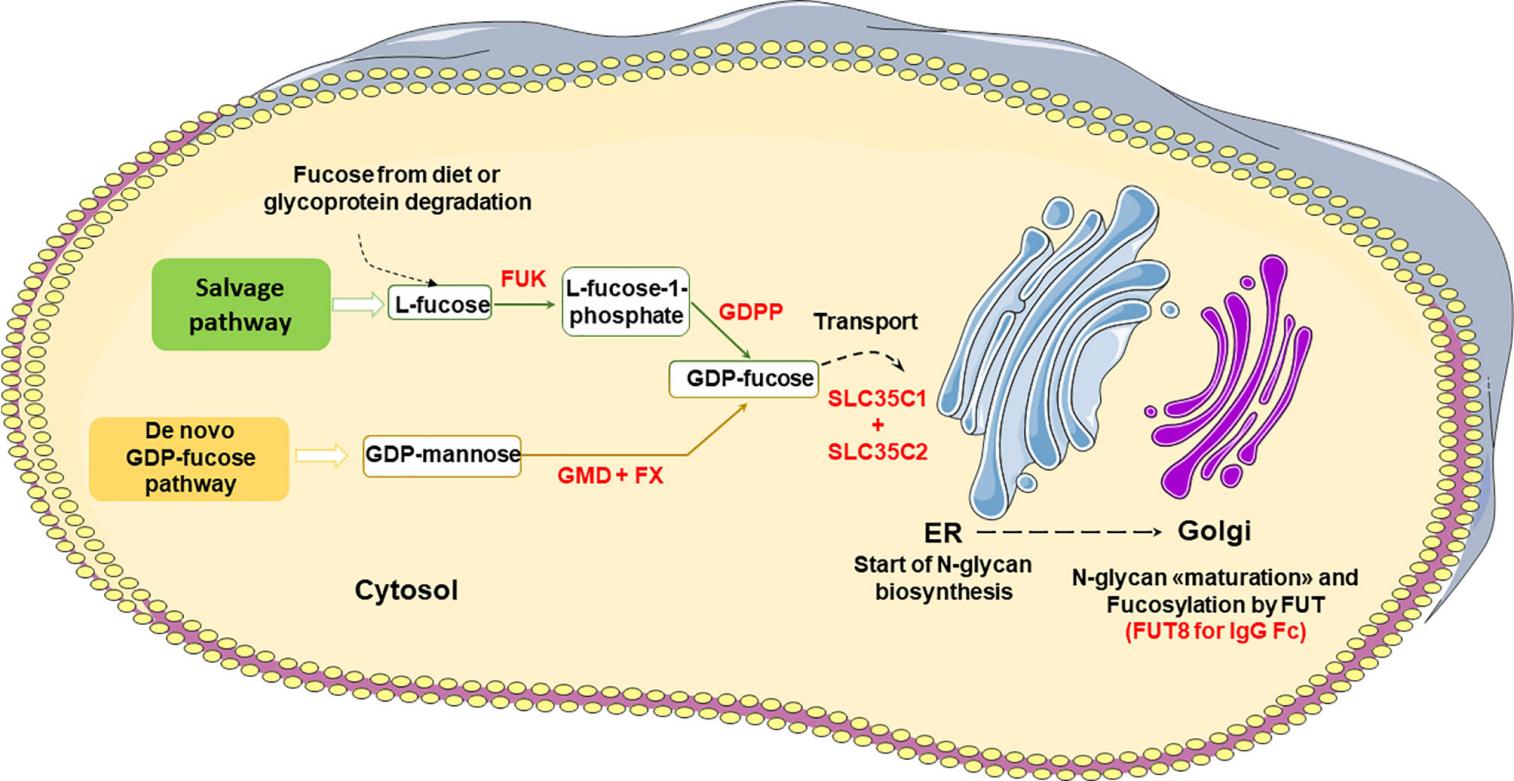
Major pathways of GDP-fucose biosynthesis and IgG Fc core fucosylation. FUK, Fucose Kinase; GDPP, GDP-fucose-pyrophosphorylase; GMD, GDP-mannose 4,6 dehydratase; FX, GDP-4-keto 6-deoxymannose 3,5-epimerase-4-reductase; FUT, Fucosyltransferase.

The enzymatic pathways for GDP-fucose biosynthesis and Fc core fucosylation are shown in **Figure 3** (26, 30, 36). L-Fucose (6-deoxy-L-galactose) is a monosaccharide obtained by glycoprotein degradation or diet. In the salvage pathway, L-fucose is phosphorylated in the cytosol to fucose-1-phosphate by fucokinase (FUK), and then converted to GDP-fucose by GDP-fucose-pyrophosphorylase (GDPP), an essential substrate for the fucosylation of proteins (**Figure 3**). Alternatively, and most commonly, *de novo* GDP-fucose is synthesized from GDP-mannose by GDP-mannose 4,6 dehydratase (GMD) and then GDP-4-keto 6-deoxymannose 3,5-epimerase-4-reductase (FX) (**Figure 3**). GDP-fucose is transported to the ER *via* the SLC35C1 and SLC35C2 transporters and used by several fucosyltransferases (FUT) in the Golgi to fucosylate glycoproteins. There are 11 different FUTs, but only FUT 8 catalyzes IgG Fc core fucosylation *via* an α-1,6 linkage (**Figure 3**). As mentioned above, the presence of bisected GlcNAc inhibits the addition of core α-1,6 fucose (26, 30, 36).

These pathways show that there are multiple steps where a modulation of fucosylation can take place including the supply of diet fucose (36). The mechanisms of IgG Fc glycosylation regulation in B cells in healthy individuals and in disease are however still little understood.

## Strategies to Generate Therapeutic IgG1 With Low Levels of Fucose Levels

In the last 10-15 years, removal of IgGs Fc core fucose has been shown to be an important method to enhance ADCC by therapeutic IgG1 mAbs (37–39). Based on the knowledge of the pathways of N-glycan fucosylation described above, several strategies have been used to generate therapeutic IgGs with low or no fucose [reviewed by Pereira et al. (40)]. These are briefly summarized below:

i. The selection of cell lines that naturally have low FUT8 enzyme, such as the Y2B/0 rat cell line (38).

ii. The creation of FUT8 knock out cell lines such as CHO FUT8^-^/^-^ (41, 42). These produce antibodies completely lacking fucose, but may have a low growth rate unless specifically adapted.

iii. The selection of natural cell lines with defects in other enzymes involved in fucosylation, such as Lec 13, which has defective GDP mannose 4,6 dehydratase (GMD) which converts GDP mannose to GDP-fucose, the substrate for N-glycan core fucosylation (**Figure 3**) (37, 43).

iv. The creation of engineered cell lines lacking GMD, GDP-L-fucose synthase (FX) and/or the GDP-fucose transporter SCL35C1 (**Figure 3**) (44, 45). For example, the FX^-^/^-^ and GMD^-^/^-^ CHOZN^®^ cell clones produced IgG1 antibody with core fucosylation reduced to 6-8% or 1-3%, respectively. The FX^-^/^-^ clones also showed some aberrant glycan forms suggesting the GMD^-^/^-^ CHOZN^®^ cell line may be the best choice (44).

v. Another strategy is to engineer CHO clones with higher β-1,4-N-acetylglucoseaminyl-transferase III (GnTIII), best with a Golgi localization domain (2, 31, 46, 47). GnTIII catalyzes the transfer of GlcNAc to a core mannose residue in N-linked oligosaccharides *via* a β-1,4 linkage, which results in the formation of a bisected sugar chain. The bisection GlcNAc inhibits the transfer of fucose by FUT8, so that GnTIII overexpressing cells produce antibodies with lower levels of core fucose.

vi. Another modality is to use non-mammalian cells such as plant or insect cells that are engineered to synthesize human-type N-glycans, to reduce potential immunogenicity, but lack core fucosylation capacity (48).

vii. Cells can be treated with inhibitors of FUT8 such as 2F-Peracetyl-Fucose (49) or anti-FUT8 antibody (50).

viii. Finally, antibodies can also be enzymatically modified *in vitro* by treatment with glycosidase and glycosynthase enzymes. This is somewhat more complex since it requires antibody deglycosylation followed by a controlled glycosylation (51, 52).

The major strategies based on engineered mammalian cell lines are listed in **Table 1**.

**Table 1:** Examples of cell lines and strategies developed for low fucose antibody production.

Cell line or system	Enzyme defect	Approximate Fc core fucosylation level (normal is >90%)	References
Lec 13 (CHO mutant)	Defective GMD	10%	(37, 43)
YB2/0 (rat)	Low FUT 8	9-30%	(38)
CHO FUT8^-^/^-^ (e.g. Potelligent^®^)	FUT8 KO	0%	(41, 42)
CHO GMD^-^/^-^GFT^-^/^-^	GMD+SLC35C1 KO	0%	(45)
CHO FX^-^/^-^	FX KO	6-8%	(44)
CHO GMD^-^/^-^	GMD KO	1-3%	(44)
CHO GnTIII^+++^ (e.g. GlycomAbs^®^)	GnTIII overexpression	10-15%	(46, 47)

FUT, Fucosyltransferase; GMD, GDP-mannose 4,6 dehydratase; FX, GDP-4-keto 6-deoxymannose 3,5-epimerase-4-reductase; GnTIII, N-acetylglucosamine transferases III; SLC35C1, GDP-fucose transporter; KO, knock out.

It is worth noting that, depending on the system used, the amount of fucose may vary from 0% for cell lines lacking FUT8 enzyme to 10-30% for the those with reduced FUT8 or other enzymatic modifications (**Table 1**). Also, different production cell lines and systems may lead to antibodies with different N-glycan profiles, not only regarding fucose residues. These may in turn affect function of antibodies since carbohydrates other than fucose, for example galactose or sialic acid may affect CDC or inflammation, respectively, among others (33, 53). A more detailed description of the role of galactosylation and sialic acid is beyond the scope of this review.

## Functional Consequences of Low Core Fucose on IgG1 Asn 297 N-Glycan

### Binding to FcγRIIIA and FcγRIIIB

Human, humanized or chimeric IgG1 antibodies lacking fucose on the Asn N-glycan bind with 10-100 fold higher affinity to human FcɣRIIIA and FcɣRIIIB (CD16B) (37, 54, 55). Structural studies have shown an increased carbohydrate-carbohydrate interaction between the N-glycans of FcγRIIIA and Fc, explaining the higher affinity of afucosylated IgG1 (56, 57).

### NK Cells and ADCC

Since FcɣRIIIA is the major activating receptor on NK cells and mediates ADCC, the net result of increased binding to FcγRIIIA is a significant enhancement of ADCC by afucosylated IgG1 antibodies with respect to their fully fucosylated counterpart (2-40 fold, also depending on galactosylation) (37–39) (**Table 2**). In addition, FcɣRIIIA has relatively low-medium affinity for IgG so that ADCC is inhibited by excess IgG in plasma. In contrast, ADCC induced by afucosylated IgG1, which has a significantly higher affinity for FcɣRIIIA, is not significantly inhibited by plasma IgG. Thus afucosylated anti-CD20 antibody may be more effective in inducing ADCC in whole blood by 2 mechanisms: 1) higher affinity for FcɣRIIIA and 2) significantly reduced inhibition by serum IgG (99). Afucosylated IgG1 has also been reported to induce greater FcɣRIIIA downmodulation from the NK cell surface, a phenomenon which takes place *via* shedding of the extracellular domain by the ADAM 17 metalloproteinase and may participate in the serial target cell killing by NK cells (100).

**Table 2:** Selected therapeutic antibodies with low or no fucose, approved by FDA/EU or in clinical development.

Antibody name (code)	Antigen	Antibody isotype	Method of defucosylation	% fucose	Diseases	Major findings	references
**1.1. Approved antibodies (FDA/EU)**							
Obinutuzumab (GA101)	CD20	Humanized IgG1	CHO overexpressing GnTIII (GlycoMAb)	About 15%	B-NHL	Higher ADCC by NK and ɣδ Tells, more effective than RTX *in vivo* in some mice models or in primates	(47, 55, 58–62) (63)
						Phase III in CLL compared to RTX in combination with CLb	(64)
						Phase III studies with different chemotherapy regimen in CLL and compared to RTX	(65)
						Phase III studies in diff chemo combinations compared to RTX in untreated FL.	(66)
Mogamulizumab (KW-7061)	CCR4	Humanized IgG1	CHO FUT8 ^-^/^-^ (Potelligent)	0%	Cutaneous T cell lymphoma	Equivalent ADCC by afucosylated mAb, but with 10-fold lower antigen expression on target compared to fucosylated mAb	(67, 68)
Inebilizumab (MEDI 551)	CD19	Humanized IgG1	CHO FUT8 ^-^/^-^ (Potelligent)	0%	Neuromyelitis optica	Increased ADCC *in vitro*. Depletes B cells more effectively than fucosylated antibody in hCD19 transgenic mice (PB, spleen and BM)	(69–72)
Benralizumab (MEDI-563)	IL-5Rα	Humanized IgG1	CHO FUT8 ^-^/^-^ (Potelligent)	0%	Severe asthma with eosinophilia	Increased ADCC *in vitro*. Efficacy in depleting eosinophil in non-human primates and in clinical Phase III studies	(73–75)
Margetuximab (MGAH22)	HER2	Chimeric IgG1	CHO FUT8 ^-^/^-^ Also mutation in Fc to decreased CD32B binding	0%	Advanced metastatic HER2^+++^ breast cancer	Increased ADCC. *In vivo* increased activity in hFcɣRIII+ mice. Phase III trial in breast cancer compared to trastuzumab	(76, 77)
Belantamab vedotin (GSK2857916)	BCMA	IgG1-MMAF ADC	CHO FUT8 ^-^/^-^	0%	Multiple myeloma	Increased ADCC of naked mAbs. Phase II ORR 31%. 72% survival at 6 months	(78)
Amivantamab (JNJ-61186372)	EGFRxMET	Humanized bispecificIgG1	Low fucose producing cell line	<10%	Non-small cell lung cancer (NSCLC)	Increased ADCC, not ADCP compared to high fucose variant. Phase III NSCLC	(79)
**1.2. Selected antibodies in clinical studies**							
Ublituximab (Emab-6)	CD20	Chimeric IgG1	YB2/0	24%	CLL, B-NHL, multiple sclerosis, neuromyelitis optica	High ADCC and ADCP (not compared with fully fucosylated antibodies). Phase I and II trials in B-NHL, CLL and autoimmune diseases + neuromyelitis optica. Phase III in CLL with or w/o ibrutinib (ORR 85% vs 65%)	(80–82)
Tomuzotuximab (cetuGEX)	EGFR	Humanized IgG1 (cetuximab seq)	Glyco Express System^®^	0%	Advanced carcinoma	Increased ADCC *in vitro*, Phase I study	
						Phase II study comparing CetuGEX with cetuximab combined with chemo: no difference observed	(83)
Imgatuzumab GA201 (RG7160)	EGFR	Humanized rat IgG1 (ICR62)	CHO stably expressing GnTIII (GlycomAb)	15%	Carcinoma	Increased ADCC *in vitro*. Higher efficacy in mouse models (SCID beige or SCID hFcɣRIIIA tg) also in combination with chemotherapy	(84)
						Phase I study in EGF^+++^ solid tumors	(85)
						Open label study in advanced CRC. Decrease NK post treatment in PB	(86)
						Enhanced ADCC *in vitro*	(87)
						Favorable combination of GA201 and chemo *in vitro* and in carcinoma models in SCID mice	(88)
						Open label study of GA201 vs cetuximab in head & neck squamous carcinoma (N=44). Greater decrease in NK in PB and greater cytokine release with GA201 vs CTX. No difference in clinical response.	(89)
KHK4083	OX40	Human IgG1	FUT8 ^-^/^-^Potelligent	0%	Ulcerative colitis	Phase I	(90)
Tragex	HER2	Humanized IgG1	Glyco Express system^®^ (FUT8-/-)		HER2+++ tumors	Increased ADCC *in vitro*. Enhanced activity *in vivo* in hFcɣRIIIA tg mice. Phase I in HER2^+++^ solid tumors	(37, 91–94)
Cusatuzumab (JNJ-74494550, ARGX-110)	CD70	Humanized IgG1	CHO FUT8 ko (Potelligent)	0%	Hematological and solid cancers	Phase I study	(95, 96)
Bemarituzumab (AMG 522)	FGFR2b	Humanized IgG1	CHO FUT8	0%	Gastric cancer FGFR2b^+++^	Increased ADCC *in vitro*. Efficacy *in vivo* in mouse sc SCID model. Recruitment of NK and T cells into tumor.	(97, 98)

ADCC, Antibody dependent cellular cytotoxicity; ADCP, Antibody dependent cellular phagocytosis; BM, Bone marrow; B-NHL, B-Non Hodgkin’s lymphoma; CLb, chlorambucil; CLL, Chronic lymphocytic leukemia; CR, Complete response; EGFR, Epidermal growth factor receptor; FL, follicular lymphoma; PB, Peripheral blood; NSCLC, Non-small cell lung cancer; ORR, Overall response rate; RTX, Rituximab; SCID, Severe combined immunodeficient.

### Phagocytosis by Macrophages

FcɣRIIIA is expressed by monocytes/macrophages, particularly M2 and red pulp macrophages as well as microglial cells. However, macrophages also express the activating FcɣRI and FcɣRIIA, and FcγRI is thought to be the major mediator of phagocytosis of IgG1 opsonized targets (10, 11, 101). Therefore, afucosylated therapeutic mAbs, despite binding with higher affinity to FcɣRIIIA, are not generally reported to significantly enhance phagocytosis, at least *in vitro* (10, 11, 102). There is some evidence that phagocytosis of targets opsonized with anti-CD20 by liver Kupfer cells *in vivo* is enhanced by the afucosylated mAb (103), but this point still needs to be further investigated and confirmed. In the context of polyclonal alloimmune anti-HPA1a IgG1 antibodies (see below), increased phagocytosis of target platelets *in vitro* by both monocytes (*via* FcɣRIIIA) and PMN (*via* FcɣRIIIB) has been reported (104).

### ADCC by ɣδ T Cells

FcɣRIIIA is also expressed by ɣδ T cells and these can mediate ADCC in presence of IgG1 antibodies (105, 106). There are reports of increased ADCC by ɣδ T cells in presence of low fucose anti-CD20 mAb obinutuzumab compared to rituximab (58, 107). ɣδ T cells represent <5% of T cells in the circulation of healthy individuals; they are also localized in non-lymphoid tissues and constitute the majority of immune cells in some epithelia (108). The role of ɣδ T cells in the response to IgG1 antibody *in vivo* is not well understood.

### ADCC and Trogocytosis by PMN

IgG1 with low Fc core fucose also binds more strongly than fucosylated antibody to FcɣRIIIB, which has >97% sequence identity with FcɣRIIIA in its extracellular IgG binding domain (14, 15, 55). FcɣRIIIB is a GPI-linked receptor lacking activating module (ITAM), is expressed only by PMN and at high levels on these cells (5, 55, 109). Low fucose anti-CD20 therapeutic antibody obinutuzumab was shown to activate PMN more effectively than rituximab, which is ≥90% fucosylated, initially suggesting that enhanced FcɣRIIIB binding was responsible for this effect (55). However, subsequent experiments performed with PMN isolated from a rare FcɣRIIIB null donor, as well as the observation that PMN may express very low levels of FcɣRIIIA, indicated that PMN activation by afucosylated anti-CD20 antibodies may be mediated by FcɣRIIIA, FcɣRIIIB, or both, depending on conditions (110). The presence of low levels of FcγRIIIA on resting or activated PMN however still needs to be confirmed. The activation of PMN by anti-CD20 antibodies does not induce ADCC or phagocytosis, but only trogocytosis and cytokine production (55, 110). Interestingly other antibodies, such as anti-EGFR antibodies do mediate ADCC of tumor targets by PMN, but this function strictly requires FcɣRIIA (15). Furthermore, PMN and FcɣRIIA mediated ADCC is inhibited by FcɣRIIIB (111, 112). Indeed, ADCC by PMN is diminished in the presence of afucosylated anti-EGFR, because the latter binds more strongly to FcɣRIIIB, which is highly abundant on PMN and is thought to compete with FcɣRIIA. Therefore, at least in this context, FcɣRIIIB may act as a decoy receptor (111, 112). It remains to be established whether and how the level of fucosylation of other antibodies, for example those directed against microbes, affect PMN functions.

### Cytokine Release by Immune Cells

Several studies indicate that afucosylated IgG1 antibodies induce a more rapid and intense cytokine release by NK cells, monocytes/macrophages, PMN and/or ɣδ T cells compared to their fully fucosylated counterparts. Induced cytokines include IFN-ɣ, MCP1, IL-6, TNF, MIP1αβ, Rantes, IL-8 (55, 100, 107, 113–115). The level of cytokines induced *in vitro* is however generally quite low and the significance of such release *in vivo* is not fully clear. Nonetheless, the more frequent or severe immediate reaction syndrome observed in patients treated with obinutuzumab compared to rituximab indicates that increased cytokine release, particularly IL-6 and IFN-ɣ by afucosylated antibodies may be relevant *in vivo* (64, 116, 117). Cytokine release should therefore be carefully studied during the pre-clinical and clinical development of afucosylated antibodies.

**Figure 4:**
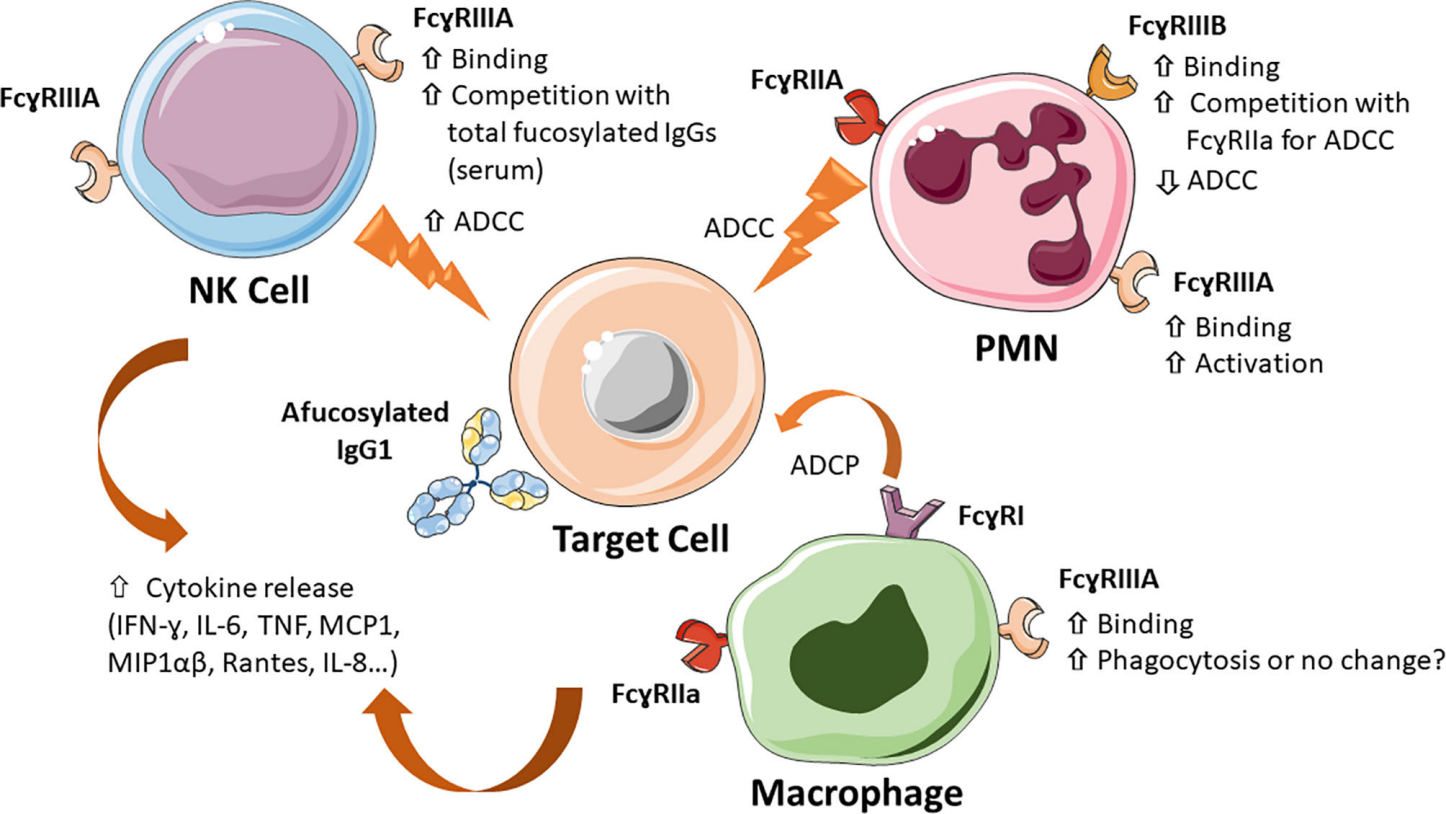
Major mechanisms of action of Fc core afucosylated IgG1 antibodies. IgG1 antibodies lacking Fc core α-1,6-fucose show a 10-100 fold increased binding to FcɣRIIIA and FcɣRIIIB on the indicated immune cells (NK, monocytes/macrophages and PMN), which results in increased ADCC by NK cells, enhanced competition with plasma IgGs, increased PMN activation, increased inhibition of FcγRIIA-mediated ADCC by PMN, induced by some antibodies (e.g. anti-EGFR mAbs). The effect of monocyte/macrophage induced ADCP is less clear. Low Fc core fucose can also increase release of cytokines, such as IL-6, TNF-α and IL-8, both *in vitro* and *in vivo*.

The functional effects of IgG1 Fc core afucosylation are summarized in **Figure 4**.

## Evidence That Afucosylation Enhances the Efficacy of Therapeutic mAbs *In Vivo* in Animal Models

Studying the role of Fc core fucosylation using small animal models is complicated by the fact that mice express different set of FcɣRs compared to humans (5, 118–120). The more recently discovered mouse FcɣRIV molecule has been shown several years ago to be the murine ortholog of human FcɣRIIIA and to be an important mediator of human IgG1 efficacy in mice. Afucosylated human IgG1 binds with higher affinity also murine FcɣRIV (121). This suggests that, despite species differences, human IgG1 therapeutic antibodies can be tested in mice, at least for some aspects of their functions. However, because of differences in FcɣR expression pattern in mice, humanized mice or human FcɣRIIIA transgenic mice may be used more appropriately. Nonetheless one should bear in mind that mice do not have the equivalent of FcɣRIIIB, which therefore makes this species not completely adequate to test the role of PMN in antibody efficacy *in vivo*, unless fully humanized models are used (119). Despite these caveats, it is worth noting that afucosylated or low fucose anti-cancer mAbs have consistently been shown to control tumor growth more efficiently than their fully fucosylated parent molecules in mice (69, 84, 92, 122), as well as macaque, the latter having a more similar pattern of FcɣRs to humans (123). These results have led to an increased attention to the N-glycan profile of therapeutic mAbs (124), as well as the development of new afucosylated therapeutic antibodies in a variety of clinical contexts. These are discussed below.

## Anti-Cancer mAbs With Low Core Fucose in the Clinic

Many antibodies with no or low Fc core fucose have entered pre-clinical or clinical development to treat different diseases. Seven such antibodies have been approved so far by FDA/EMA (**Table 2**). They include one bispecific and one ADC, the others being unconjugated IgG1 antibodies. Five are approved for cancer therapy and two for immune/inflammatory diseases. Many other antibodies are in clinical trial (**Table 2** **, part 2**) or in pre-clinical development (47, 55, 58–79). In all cases, a significantly enhanced ADCC activity by NK cells has been shown *in vitro* and, in some, more effective target depleting activity *in vivo* in mice or non-human primates, as discussed above (**Table 2**).

It is obviously difficult to evaluate whether removal of Fc core fucose significantly increases the efficacy of therapeutic anti-cancer mAbs in patients without performing phase III comparative studies of parent and afucosylated counterpart, studies that are rarely performed. Glycoengineered anti-CD20 antibody obinutuzumab was the first therapeutic antibody with low fucose to be approved in 2013 for the treatment of B-cell neoplasia. It has been extensively compared with rituximab *in vitro* and in clinical trials in Phase III studies in chronic lymphocytic leukemia and B-NHL, usually in combination with chemotherapy. Obinutuzumab has shown either enhanced activity or, in some cases, non-inferior activity to rituximab depending on disease contexts and treatment protocols (64, 116, 125). However, these two anti-CD20 antibodies differ from each other in aspects other than level of core fucosylation. They differ in epitope recognition, binding mode to CD20, and to some extent mechanisms of action (125). Rituximab induces higher CDC whereas obinutuzumab induces homotypic adhesion and direct cell death, in addition to the higher ADCC due to low fucose (62, 102, 126). It is therefore difficult to clearly identify the mechanism of higher efficacy of obinutuzumab in some disease contexts, compared to rituximab.

Another way to understand whether FcɣRIIIA binding affinity (and therefore the degree of IgG1 core fucosylation) is important for antibody efficacy is to study the response of patients carrying different FcɣRIIIA variants, in particular the 158V (high affinity) and 158F polymorphism (lower affinity). The studies of rituximab activity in follicular lymphoma patients treated with rituximab as single agent and the correlation between clinical response and the FcɣRIIIA-158V carriers suggested that FcɣRIIIA and ADCC by NK cells are important for the *in vivo* efficacy of this antibody (127). Similar data have been obtained in HER2 overexpressing breast cancer patients treated with trastuzumab (128). Nonetheless, for both rituximab and trastuzumab, these data have not been confirmed in all clinical contexts, perhaps also due to the fact that these therapeutic mAbs are generally used in combination with chemotherapy, which itself may modulate cell-mediated mechanisms (9, 129). Thus, both FcγRIIIA-dependent and independent mechanisms are likely to be important for anti-cancer IgG1 efficacy and these may vary in different clinical and therapeutic conditions.

Importantly, defucosylation has been shown not to alter significantly FcRn binding or pharmacokinetics *in vivo*, encouraging the development of afucosylated antibodies for clinical use (30).

To summarize, the fact that afucosylated IgG1 have consistently shown higher FcɣRIIIA binding and ADCC *in vitro* as well as often a greater efficacy *in vivo* in animal models without significant changes in antibody pharmacokinetics *in vivo* (69, 92) has led many groups to design and generate afucosylated therapeutic IgG1 mAbs or BsAb, as well as ADCs.

## Modulation of IgG Fucosylation in Physiological States and Disease

### Physiological Modulation of Fc N-Glycan Fucosylation

The glycosylation pattern of IgGs has been shown to be modulated during life. Children starting to produce their own IgGs show higher N-glycan core fucose levels, which decrease during childhood (130, 131). In adults, the levels of IgG fucosylation is quite stable in absence of pathology or interventions, but quite variable between individuals (ranging from 1.3 to 19.3% afucosylation), presumably due to hereditary as well as environmental factors (29).

How changes during pregnancy or aging are brought about is not clear. During pregnancy, chorionic gonadotropin (hCG) has been shown to promote the development of IL-10 producing B cells which are associated with production of highly core fucosylated IgG (132). The B cell response to infections that induce afucosylated IgGs probably explains the increased afucosylation during childhood (see below), but the mechanisms are unclear. Recently the spleen has been suggested to be an important site of fucosylation regulation: IgG1-Fc core fucosylation was observed to be increased in trauma splenectomized healthy individuals as well as in spherocytosis or immune thrombocytopenia (ITP) patients that were splenectomized, compared to non-splenectomized controls (133). There are several possible explanations for this observation: 1) The spleen may be a site of afucosylated IgG generation. Indeed in ITP, the spleen is also a source of anti-platelets IgGs which are highly afucosylated compared to total IgGs. 2) The spleen removes preferentially immune complexes containing afucosylated IgGs *via* FcɣRIIIA positive myeloid cells, so that splenectomy induces a change in the ratio of fucosylated/afucosylated IgGs (133). Further investigation of these possible mechanisms is warranted.

Several gene array analyses correlating IgG N-glycan traits with genome-wide polymorphic variants have allowed to identify gene pathways involved in N-glycome regulation, several of which were confirmed in more than one study (134–140). Some of the genes identified are glycosyltransferases, most significantly FUT8, B4GALT1 and MGAT3 (the latter encoding the GnTIII enzyme)(**Figure 2**), as well as transcription factors known or suspected to regulate these genes, such as RUNX1, RUNX3, IKZ1, IKZ3, IRF1, SMARCB1, TBX21O and HFN1 (138). Others are novel genes with no known role in glycosylation (135). With specific regard to fucosylation, FUT8 itself, the fucosidase FUCA2 and transcription factors regulating FUT8, most notably IKZF1, have been implicated in several studies (135, 137, 141). Altogether these results suggest that the expression level, localization and perhaps substrate availability of glycosylation enzymes define IgG glycosylation patterns. Clearly, further work will be required to fully identify the pathways involved and their role in the physiological regulation of IgG Fc N-glycan composition and core fucosylation in B cells.

Since N-glycosylation and core fucosylation take place in the ER and Golgi (**Figures 2** and **3**), the physiological state, pH, ionic and redox of ER and Golgi can also affect glycosylation (142, 143). Indeed some studies have evidenced the role of ER stress in modifying protein glycosylation patterns (144). In particular in B cells, the mutation or deletion of the ER protein Jagn1 leads to ER stress, reduced IgG core fucosylation and increased sialylation, indicating that afucosylated IgG1 may be induced during ER stress (145).

Interestingly many loci identified to play a role in regulating the glycosylation pattern of IgGs and other plasma proteins are also associated with autoimmune and inflammatory diseases (135, 137). The specific role of IgG Fc core fucosylation in these pathological conditions is discussed below.

### IgG1 Fc Core Fucosylation in Alloimmunity

The fact that IgG Fc core fucosylation is physiologically modulated was first observed in the context of alloimmunization, frequently occurring during pregnancy, and mostly involving red blood cells (RBC) and platelets. Rhesus D antigen alloimmunization during pregnancy and delivery of a RhD^+^ fetus by a RhD negative mother has been in the past the most common cause of hemolytic disease of the fetus and newborn (HDFN), occurring in sensitized mothers who had given birth to a previous RhD^+^ baby. The disease is due to anti-RhD IgG antibodies crossing the placenta and destroying the RhD^+^ RBC in the reticuloendothelial system of the fetus and newborn, causing severe fetal anemia and hydrops fetalis in severe cases. Before the advent of prophylaxis of RhD negative women with anti-RhD immunoglobulin, which prevents allo-immunization, HDFN affected 150 per 100 000 births and 10% of perinatal deaths in the Caucasian population. Analysis of IgG glycosylation in alloimmunized pregnant mothers has revealed a variable decrease in IgG1 Fc core fucosylation (even down to 12%), whereas the total IgG remained highly fucosylated, as in healthy individuals (>90%). Furthermore, the level of anti-RhD Fc core fucosylation correlated significantly with FcγRIIIA-mediated ADCC and with low fetal-neonatal hemoglobin levels (146).

A later *in vitro* study investigated the functional activity of monoclonal anti-RhD antibodies of different subclasses (IgG1, 2, 3 or 4), bearing either low (usually <30%) or high (>90%) Fc core fucose levels (147). Whereas reduced fucosylation increased FcɣRIIIA binding of all IgG subclasses, only defucosylated IgG1 and IgG3 showed a 12- and 7-fold increase in ADCC by NK cells, respectively, presumably due to the fact that IgG2 and IgG4 are poor binders of FcɣRIIIA (147). Afucosylation of the anti-RhD mAbs did not enhance *in vitro* phagocytosis of RBC by GM-CSF (M1) or M-CSF (M2) induced macrophages, reminiscent of what has been most often observed with anti-tumor antibodies (see above). Another study of an anti-RhD mAb with low core fucose showed a higher RBC clearance capacity *in vivo* in mice, compared to highly fucosylated antibody (148).

These data altogether show that IgG Fc core fucosylation is likely to be an important pathological feature in HDFN with diagnostic potential, and that a significant mechanism of the disease may be RBC destruction by NK cells (149), in addition to ADCP by the reticuloendothelial system.

RBC alloantigens other than Rhesus D (c, E, Kell) can induce an antibody response and HDFN. The level of defucosylation of anti-Kell, but not anti-c or anti-E, was shown to correlate with severe fetal anemia. These data suggest that the antigens shape the type of response (150).

Alloimmunization of mothers by fetal platelets can also occur during birth or pregnancy, leading to the generation of antibodies directed against polymorphic human platelet antigens (HPAs), the phagocytosis of opsonized platelets in fetal spleen and liver and the development of fetal and neonatal alloimmune thrombocytopenia (FNAIT), a rare but potentially fatal diseases of the fetus and newborn (151). FNAIT can also occur during first pregnancy of incompatible mothers and can have severe consequences, including intracranial hemorrhage. In 80% of FNAIT cases, the targeted antigen is human platelet antigen-1a (HPA-1a), i.e. a polymorphic epitope of the β3 integrin (152). The HPA-1a antigen is presented to maternal T and B cells in combination with the HLA DRB3*01:01 molecule and leads to production of anti-platelets, mostly IgG1 antibodies (151, 153). Several groups have shown that pregnant women have decreased, although variable, IgG1 Fc core fucosylation, which is specific for anti-HPA-1a as opposed to total IgG. Low core fucose on anti-platelet antibodies appeared to increase phagocytosis by monocytes and PMN (104). Reduced anti-HPA-1a Fc core fucosylation as well as antibody levels correlated with decreased neonatal platelet counts and increased disease severity in FNAIT patients (104, 152, 154). Interestingly, low fucosylation of anti-HPA antibodies was observed in patients with FNAIT but not in those with refractory thrombocytopenia following platelet transfusion, indicating that the level of fucosylation is antigen dependent and/or related to the immune milieu defined by pregnancy (104).

### IgG1 Fc Core Fucosylation in Autoimmune and Inflammatory Diseases

The IgG Fc N-glycosylation pattern of total or antigen specific IgGs has also been investigated in several autoimmune and inflammatory diseases, including rheumatoid arthritis (RA), systemic lupus erythematosus (SLE), multiple sclerosis (MS), autoimmune thyroid diseases, inflammatory bowel diseases (ICB), chronic obstructive pulmonary disease (COPD), ulcerative colitis (UC), etc. In these clinical contexts the greatest changes have been observed in galactosylation and sialylation, which in some cases such as in RA correlate with disease activity (155, 156). Fc core fucosylation has been observed to be modulated in only few autoimmune diseases, with an increase observed in RA, juvenile idiopathic arthritis and Crohn's diseases and a decrease in SLE, MS, and Lambert-Eaton myasthenic syndrome, immune thrombocytopenia (ITP) and either increase or decrease in different thyroid autoimmune diseases (157–159). It is worth noting that in this context, total IgGs rather than antigen-specific IgG have generally been analyzed. The pathological significance of these results is still unclear, except that low galactosylation and sialylation as well as high fucosylation of IgGs have been associated with a pro-inflammatory state (160). Several reviews of the state of the art regarding Fc N-glycosylation in autoimmune and inflammatory diseases has been published recently and we suggest the reader to consult these articles for further details on the subject (157, 161–163).

Interestingly, intravenous immunoglobulins (IVIGs) are extensively used as an immunomodulatory agent to treat autoimmune and inflammatory diseases. One mechanism of action of IVIGs is thought to be through competition with pathogenic IgG for Fcɣ receptors on immune cells. A very recent report analyzed the functional effects of different IVIGs glycoforms (i.e. IVIGs carrying 2 galactose, 2 sialic acids or no galactose, with or without α-1,6-fucose). Galactosylated and afucosylated IVIGs [(G2)_2_] had the highest affinity for FcɣRIIIA. The greatest effect was given by fucose removal as expected, but presence of 2 Gal residues increased affinity still further. Interestingly, (G2)_2_ IVIGs were also most potent in blocking ADCC *in vitro* and in reducing inflammation and serum IL-6 levels in a collagen antibody-induced arthritis model in mice (164). These data confirm that the anti-inflammatory activity of IVIGs is in part mediated *via* blockade of FcɣRIIIA by their galactosylated, non-fucosylated IgG component, at least in some clinical settings (165). They also corroborate the immunomodulatory capacity of Fc N-glycan composition in health and disease, particularly regarding core α-1,6-fucose and galactose. Finally, they suggest that modified IVIGs [(G2)_2_] may be a more effective immunomodulatory agent than unmodified IVIGs. This hypothesis will need to be verified.

### IgG1 Core Fucosylation During the Humoral Response to Infectious Agents

Evidence for an important role of antibodies in the control viral or parasitic infections has gained strength over the last decade. IgG1 antibodies in infection, just like for anti-cancer mAbs, can act through a variety of mechanisms: *via* Fab they can neutralize viruses and parasites by blocking cellular entry and the replication cycle; through Fc they induce complement activation and phagocytosis by macrophages or PMN of opsonized immune complexes, clearing the infectious agent, as well as activate ADCC by NK cells which will destroy infected cells (166–170). Fab- and Fc-mediated activities probably synergize with each other, at least in some cases, although some antibodies under some circumstances can also enhance infection probably by facilitating the entry of the virus into FcɣR positive target cells, a phenomenon called antibody dependent enhancement (ADE). This has been particularly well described following secondary infection by a different Dengue virus serotype (166, 167, 171–173).

The role of the different FcɣRs and immune cells in protection from viral or parasitic diseases is not fully established, also because establishing suitable models is particularly challenging. Nonetheless FcɣRs as well monocytes and macrophages have been implicated as important effectors in controlling viral diseases caused by Influenza A virus, SARS-CoV and SARS-CoV-2 viruses, Chikunguya virus, West Nile virus and Yellow Fever virus (166, 170, 172, 174). Less frequently a role of NK cells or NKT cells has been demonstrated (166).

Interestingly, recent technical improvements in dissecting glycan structures have allowed to demonstrate that during natural antibody responses to viral or parasitic infections, there may be in some cases a decreased Fc core fucosylation of antigen-specific IgGs. **Table 3** lists the major examples of this phenomenon and indicates the levels of afucosylation and duration that has been reported (whenever known). IgG1 antibodies against cytomegalovirus (CMV) (175) or against the plasmodium falciparum erythrocyte membrane antigen 1 (pfEMP1), a parasite antigen expressed on infected erythrocytes (183), show the highest levels of afucosylation following infection (median 30-75%), although this was highly variable between individuals. The effect appeared to be quite stable during time, perhaps due to continuous stimulation and/or formation of long-lived plasma cells secreting these afucosylated antibodies (175, 183). Other infectious agents, such as SARS-CoV-2 virus (175–179, 184), Dengue virus (180–182), Hepatitis B virus and Mump virus (175), induce lower (median 12-18%), although significant, levels of afucosylated antigen-specific IgG1s (**Table 3**). Worth noting is that afucosylation has been demonstrated to be specific for the IgG1s that recognize the viral or parasite membrane antigen and has not been observed in total circulating IgG1 or IgGs. The mechanism involved is not yet clear. Nonetheless, present evidence suggests that antigens presented in the context of host cell membranes may induce specific Fc core afucosylation. Indeed, infection by enveloped viruses or by plasmodium falciparum decreases Fc core fucosylation of IgGs induced by membrane associated antigens, whereas vaccination with the soluble antigens from the same microorganisms does not (175, 183).

**Table 3:** Examples of modulation of Fc core fucosylation of antigen-specific IgGs in infectious diseases.

Antigen	Afucosylation level	% afucosylation(median)	IgG involved	Time frame	Clinical significance of afucosylation	ref
CMV	Low	35%	IgG1	Stable over time	Not known	(175)
SARS-CoV-2 Spike	Fast upon sero-conversion	12-18%	IgG1	Reversible	Correlates with ARDS, IL-6 and CRP levels, correlates with IL6 induction by macrophages *in vitro*	(175–179)
Dengue E and NS1 proteins	Induced by infection (1° and 2°)	12-18%	IgG1	Stable over time	Correlates with disease severity; correlates with low platelets and RBC	(180–182)
HIV-1	low	12%	IgG1	Stable over time	Unknown	(175)
HBV	Low	16%	IgG1	–	Unknown	(175)
Mumps virus	Low	12%	IgG1	–	Unknown	(175)
Plasmodium falciparum EMP1	High	30-75%	IgG1	Stable w/o antigen boost, further decreases with multiple exposure	Induces higher degranulation of FcɣRIIIA^+^ cell line *ex vivo*	(183)

ARDS, Acute Respiratory Distress Syndrome; CRP, C Reactive Protein; CMV, cytomegalovirus.

A recent paper investigating total plasma IgG N-glycosylation patterns in patients with asymptomatic filariasis (MF^+^) or with chronic disease (CP), compared to non-infected local control donors showed that CP patients had higher N-glycan heterogeneity. Afucosylation was highest in the CP group and lowest in MF^+^ group compared to controls. Other changes in glycosylation were observed, which may also have affected the immune and inflammatory state of the patients (185). Further studies will be required to investigate N-glycosylation pattern also on antigen-specific IgGs in the context of filariasis.

An important question is the functional effect of an increased level of antibody afucosylation during viral or parasitic infections and whether modulation of Fc core fucosylation is important for the control of the infecting microorganisms. This is obviously a difficult question to answer, also because afucosylation may be accompanied by other modifications in glycan structure and because glycosylation of other proteins could also be modified during infection (175, 186). Fc core fucosylation also affects the B cell receptor assembly (BCR) and BCR signaling (32). There is however some evidence that Fc core afucosylation may help in the control of infection, since the humoral response, ADCC and NK cells can play a role on the control of viral spread (187–190). Nonetheless afucosylation may also lead to major side effects, through pro-inflammatory mediators released by FcɣRIII expressing immune cells (see paragraph 5.5). Indeed, a correlation has been observed in some studies between degree of Fc core afucosylation and acute respiratory syndrome (ARDS) and inflammatory markers following SARS-CoV-2 virus infection (175, 177). Pongracz et al. confirmed decreased core fucosylation of anti-Spike protein in Covid-19 hospitalized patients, but could not observe a difference between those recovered or not in intensive care unit (178). Nor did they observe a negative correlation with inflammatory markers. In another study, degree of core afucosylation appeared to correlate with younger age of hospitalized patients (179). Recent work suggests that antibody titers, modifications of the IgG1 N-glycome profile other than afucosylation and FcɣRs other than FcɣIIIA, such as FcɣIIA, may contribute to Covid-19 disease severity (177–179, 191, 192), which would be in line with the complexity of the immune response. Clearly much work still needs to be done to fully define the factors contributing to SARS-CoV-2 infection control and ARDS.

During secondary infection with Dengue virus, the degree of Fc core afucosylation correlated with disease severity and with low platelets and RBCs (180–182).

## Discussion

Afucosylation of Asn-297 N-glycan of human IgG1 is the glycan modification with the highest impact on the effector functions of therapeutic IgG1 antibodies, notably a 10-100 fold increase in affinity for human FcɣRIIIA and 2-40 fold increased ADCC by NK cells. The effect of Fc core afucosylation on phagocytosis by macrophages is less clear and may depend on context, because these cells express several other activating FcɣRs that participate in the process of phagocytosis, in addition to FcɣRIIIA. Afucosylation of IgG1 also enhances its binding affinity to human FcɣRIIIB, a major receptor on PMN. The functional effect of this on the PMN response to therapeutic antibodies is not well understood, since many data suggest that FcγRIIIB is an activating receptor for PMN, whereas others suggest that it may act as a decoy receptor in some circumstances.

The strong enhancement of ADCC by Fc core afucosylated IgG1 antibodies has led to the development of many novel therapeutic antibodies, with the idea that enhancing their activity *in vivo*. How much more effective these antibodies are *in vivo* in the clinic, compared to their fully fucosylated counterparts is still not completely clear. Despite these uncertainties, given that animal models suggest greater efficacy of therapeutic mAbs with lower Fc core fucosylation and that afucosylation does not significantly affect the pharmacokinetics of antibodies, this modification is frequently used as a method to try and enhance the efficacy of therapeutic IgG1 mAbs. Removal of fucose can also be combined with point mutations of IgG1 antibodies to further enhance their immune-mediated mechanisms of action (193).

Novel data have accumulated showing an increase in IgG1 Fc core afucosylation during the polyclonal antibody response to infections by some enveloped viruses and parasites and during alloimmunization by RBC or platelets in pregnancy. The correlation between disease severity in alloimmunization points to a more effective removal of target cells by the core afucosylated antibodies, mediated by NK cells and/or the mononuclear phagocyte system. In the case of infectious diseases, a decreased in core fucose in IgG1 antibodies directed against membrane-associated antigens has been demonstrated in several cases. A correlation with disease severity is suggested in some cases, such as SARS-CoV-2 infection, in which the modified N-glycome may indeed induce higher immune cells activation and inflammatory cytokine release. This observation is interesting in view of the more severe or frequent cytokine release syndrome reported with some therapeutic anti-tumor afucosylated antibodies compared to fucosylated antibodies, indicating that similar mechanisms may play a role in these phenomena.

These results emphasize the strong parallelism between the activities of therapeutic IgG1 mAbs and polyclonal IgGs naturally induced in health and disease. Still much remains to be investigated and clarified about the *in vivo* role of Fc core fucosylation, not only of IgG1 but also other Ig isotypes. Further studies on the physiological mechanisms of regulation of IgG core fucosylation in B cells *in vivo* is another exciting area of research, that may allow in the future to control pharmacologically this phenomenon in different pathological conditions.

## Author Contributions

JG took the lead in writing the manuscript. AA and IC have critically revised the manuscript, designed and completed the figures and tables. All authors contributed to the article and approved the submitted version.

## Funding

This work was supported by Fondazione Cariplo (Covid-Bank project). IC was supported by AIRC 5x1000 (Project n. 21147).

## Conflict of Interest

The authors declare that the research was conducted in the absence of any commercial or financial relationships that could be construed as a potential conflict of interest.

## Publisher’s Note

All claims expressed in this article are solely those of the authors and do not necessarily represent those of their affiliated organizations, or those of the publisher, the editors and the reviewers. Any product that may be evaluated in this article, or claim that may be made by its manufacturer, is not guaranteed or endorsed by the publisher.
